# Cumulative stressor exposure predicts menstrual cycle affective changes in a transdiagnostic outpatient sample with past-month suicidal ideation

**DOI:** 10.1017/S0033291724001661

**Published:** 2024-10

**Authors:** Anisha Nagpal, Jordan C. Barone, Hafsah Tauseef, Jaclyn Ross, Zach J. Gray, Katja M. Schmalenberger, Grant Shields, George M. Slavich, Tory Eisenlohr-Moul

**Affiliations:** 1 Department of Psychiatry, University of Illinois Chicago, USA; 2 Department of Psychological Science, University of Arkansas, USA; 3 Department of Psychiatry and Biobehavioral Sciences, University of California, Los Angeles, USA

**Keywords:** affective symptoms, hormone sensitivity, menstrual cycle, psychosocial stress, sex hormones, stress, suicidality

## Abstract

**Background:**

Affective responses to the menstrual cycle vary widely. Some individuals experience severe symptoms like those with premenstrual dysphoric disorder, while others have minimal changes. The reasons for these differences are unclear, but prior studies suggest stressor exposure may play a role. However, research in at-risk psychiatric samples is lacking.

**Methods:**

In a large clinical sample, we conducted a prospective study of how lifetime stressors relate to degree of affective change across the cycle. 114 outpatients with past-month suicidal ideation (SI) provided daily ratings (*n* = 6187) of negative affect and SI across 1–3 menstrual cycles. Participants completed the Stress and Adversity Inventory (STRAIN), which measures different stressor exposures (i.e. interpersonal loss, physical danger) throughout the life course, including before and after menarche. Multilevel polynomial growth models tested the relationship between menstrual cycle time and symptoms, moderated by stressor exposure.

**Results:**

Greater lifetime stressor exposure predicted a more pronounced perimenstrual increase in active SI, along with marginally significant similar patterns for negative affect and passive SI. Additionally, pre-menarche stressors significantly increased the cyclicity of active SI compared to post-menarche stressors. Exposure to more interpersonal loss stressors predicted greater perimenstrual symptom change of negative affect, passive SI and active SI. Exploratory item-level analyses showed that lifetime stressors moderated a more severe perimenstrual symptom trajectory for mood swings, anger/irritability, rejection sensitivity, and interpersonal conflict.

**Conclusion:**

These findings suggest that greater lifetime stressor exposure may lead to heightened emotional reactivity to ovarian hormone fluctuations, elevating the risk of psychopathology.

## Introduction

While most individuals assigned female at birth (AFAB) do not experience notable menstrual cycle affective change (MCAC), a prominent minority experience moderate to severe MCAC that causes distress or impairment (Gehlert, Song, Chang, & Hartlage, 2009). MCAC is caused by an abnormal *sensitivity* to normal hormone changes (i.e. hormone sensitivity) rather than by abnormal levels or trajectories of ovarian hormones (Gehlert et al., 2009; Schmidt, Nieman, Danaceau, Adams, & Rubinow, 1998). The most extensively studied form is codified in the DSM-5 as Premenstrual Dysphoric Disorder (PMDD), a chronic affective disorder with symptoms confined to the luteal phase (Epperson et al., 2012). However, MCAC transcends diagnostic categories, with patients across psychiatric disorders demonstrating *premenstrual exacerbation* (PME) of symptoms (e.g. 60% of patients with depressive disorders; Nolan & Hughes, 2022). Further, MCAC is bi-directionally associated with suicidal ideation (SI) and behavior; psychiatric patients recruited for SI are particularly susceptible to hormone-sensitive changes in affect and suicidality around menses onset (*perimenstrually*; Owens et al., 2023; Ross et al., 2024), and patients with PMDD report high levels of SI (72% lifetime, 40% current) and attempts (34% lifetime; Eisenlohr-Moul et al., 2022; Wikman et al., 2022). Despite this evidence that MCAC is both common and dangerous, little is known about who is at greatest risk for these cyclical affective changes.

Greater exposure to psychosocial stressors is a plausible risk factor for MCAC. In animals, stressor exposure leads to GABAergic and serotonergic dysregulation, such as altered GABA_A_ receptor subunit conformation and mRNA SERT expression in brain regions involved in stress-related psychiatric disorders (Bravo, Dinan, & Cryan, 2014; Everington, Gibbard, Swinny, & Seifi, 2018; Gardner, Hale, Lightman, Plotsky, & Lowry, 2009; Lages, Rossi, Krahe, & Landeira-Fernandez, 2021; Skilbeck, Johnston, & Hinton, 2018); this dysregulation has been linked to MCAC (Frokjaer et al., 2015; Hantsoo & Epperson, 2020; Marjoribanks, Brown, O'Brien, & Wyatt, 2013a; Roca et al., 2002). Human research also supports psychosocial stressor exposure as a risk factor for MCAC. In individuals recruited for at least one affective symptom of PMDD, those with a history of physical abuse (v. without) experienced a tighter link between fluctuations in progesterone (P4) and mood or interpersonal symptoms, whereas those with a history of sexual abuse (v. without) reported more pronounced estradiol (E2)-linked anxiety symptoms (Eisenlohr-Moul et al., 2016). Similarly, PMDD patients with higher childhood adversity (i.e. higher total sum of physical, sexual, and emotional abuse as well as emotional and physical neglect) exhibit stronger increases in negative affect and stronger decreases in positive affect from the follicular to late luteal phase (Nayman, Schricker, Reinhard, & Kuehner, 2023). In nonclinical samples, one study found that higher perceived stress in the previous month predicted greater premenstrual affective changes (Gollenberg et al., 2010); another found no impact of perceived stress on midluteal affect (v. all other phases) (Guevarra et al., 2023). Despite this evidence suggesting a link between stressor exposure and MCAC, particularly in clinical samples, small sample sizes and inconsistent, imprecise stress measurement limit these conclusions (Slavich, 2019).

Moreover, associations of stressor exposure and MCAC may differ based on *type* of stressors experienced and *when* stressors occurred in the life course (McLaughlin, Sheridan, & Lambert, 2014; Stenson et al., 2021). Although prior studies measure various stressors (e.g. exposure to lifetime physical abuse v. childhood adversity), none have comprehensively cataloged all major stressors across the life course, which is necessary to understand how diverse stressor exposures across developmental stages relate to MCAC.

To address this gap, we examined continuous daily cyclical affective symptoms in a large sample of transdiagnostic psychiatric outpatients, known to be more vulnerable to MCAC than a community sample (Hartlage, Brandenburg, & Kravitz, 2004; Kuehner & Nayman, 2021; Nolan & Hughes, 2022). We assessed daily symptoms and lifetime stressor exposure using the Stress and Adversity Inventory (STRAIN; Slavich & Shields, 2018). The STRAIN improves on prior studies by assessing exposure to various distinct stressors across the lifetime (Slavich, 2019). It includes stressors characterized by physical danger, ranging from personal injury to physical and sexual abuse, and interpersonal loss, ranging from parental divorce to neglect to the death of a loved one, thus capturing exposure to ‘threat’ and ‘loss’ as conceptualized in the NIMH's Research Domain Criteria (RDoC) Framework (Insel et al., 2010). These dimensions also fit the Dimensional Model of Adversity and Psychopathology framework, where ‘threat’ (experiences posing danger to physical well-being) and ‘deprivation’ (absence of expected environmental stimuli and social support) are hypothesized to have distinct effects on neural development and behavioral outcomes (McLaughlin et al., 2014).

To test hypotheses, we used cross-level interactions in multilevel models to examine whether person-level stress variables shaped day-level associations between the cycle and symptoms. First, we tested if total lifetime stressor exposure predicts steeper premenstrual affective symptom increase and slower postmenstrual recovery. Of note, participants in the study were previously included in a larger analysis demonstrating average perimenstrual symptom worsening in the sample (Ross et al., 2024). Next, we tested associations of specific stressor characteristics with cyclical symptom trajectories (pre-menarche v. post-menarche stressors, loss v. danger stressors). Based on our prior finding that physical abuse predicts stronger P4-affect coupling in PMDD (Eisenlohr-Moul et al., 2016), we hypothesized that physical danger stressors would be more strongly related to cyclical affective changes than interpersonal loss stressors. Further, since early life adversity impacts neurobiological risk for psychopathology (McLaughlin et al., 2014), we expected earlier timing of stressor exposure – e.g. pre-menarche stressors – to be more strongly associated with cyclical affective changes than post-menarche stressors.

## Materials and methods

### Preregistration

All hypotheses and planned analyses were preregistered on Open Science Framework (https://osf.io/qn4e9) prior to analysis.

### Recruitment and enrollment

Data were collected from two crossover clinical trials (NCT03498313, NCT04112368) investigating perimenstrual exogenous E2 and/or P4 effects on cyclical changes in SI and related psychiatric symptoms. Eligibility criteria included: AFAB; age 18–45; BMI 18–35; 21–35-day self-reported menstrual cycles; past-month SI; basic health insurance; and current outpatient mental healthcare. Exclusionary criteria included: exogenous hormonal medications/devices; pregnancy, breastfeeding, or childbirth within the prior 12 months, metabolic, autoimmune, neurological, gynecological, or other chronic nonpsychiatric diseases; heightened genetic risk for thromboembolism or hormone-related cancer; current nicotine use; diagnosis of PMDD using prospective ratings *or* seeking treatment for PMDD (given that evidence-based treatments for PMDD are available, and the hormone manipulations in the parent trials were not evidence-based treatments for PMDD); history of hospitalization for mania or psychosis; substance use preventing study participation; or elevated, imminent suicide risk. The study recruited a transdiagnostic sample of mental health outpatients reporting past-month SI, expecting a significant MCAC, as 60% of patients with depressive disorders demonstrate clinically-significant MCAC (Hartlage et al., 2004). No participants attempted suicide or required inpatient care after entering the study.

Enrollment included eligibility review, informed consent, Structured Clinical Interview for DSM-5 (SCID-5; First, Williams, Karg, & Spitzer, 2015), demographic surveys, and the STRAIN (Slavich & Shields, 2018). Trained research assistants administered the SCID-5 under licensed clinical psychologist supervision. Participants were enrolled continuously regardless of cycle phase. Daily surveys began the day after enrollment. Present analyses include only participants who completed the STRAIN (Slavich & Shields, 2018) and documented at least one ovulation (via urine luteinizing-hormone/LH surge ovulation test).

### Daily surveys

For one to three cycles before the parent trial's first experimental phase, and for an additional ‘washout’ cycle between experimental phases, participants completed daily surveys reporting menses, sleep, medication use, physical pain (0–10 scale), acute illness, and affective/ suicide-related symptoms. Some participants completed multiple baseline cycles due to scheduling conflicts, treatment changes, failure to document LH surge (e.g. due to missed tests), COVID-19 safety concerns, or low daily survey completion rates during the first cycle.

### Measures

#### Time coding: percent of luteal and follicular phase elapsed

Menstrual cycle time was coded based on self-reported menses and estimated ovulation. Out of 421 cycles, ovulation was confirmed in 219 cycles through LH-surge ovulation tests; each participant contributed at least one menses-to-menses cycle with a positive LH test. The remaining 202 cycles predominantly occurred when ovulation testing was not required by study protocols.

For cycles without ovulation testing, we estimated ovulation. First, we measured the duration of each cycle from the onset of one menses up to and including the day before onset of subsequent menses, for example, 32 days. Using normative data from Bull et al. (2019), which utilizes a large dataset of ovulatory cycles (over 600 000 cycles), we determined average lengths of the luteal and follicular phases for the observed cycle duration. For example, for a 31-to-35-day cycle, the average luteal length was 12.9 days (40% of the total cycle, rounded) and the average follicular length was 19.5 days (60% of the total cycle, rounded). Based on these percentages, we estimated ovulation day (mapped onto LH + 1) within each menses-to-menses cycle. In this example, ovulation would be estimated on day 19 (rounded from 19.2), after 60% of the 32-day cycle had elapsed (corresponding to the proportion of the cycle estimated to be follicular). To preserve data quality, we restricted our analysis to cycles lasting 21 to 35 days due to uncertainty of ovulation dates in shorter or longer cycles (Schmalenberger et al., 2021).

To ensure shared hormonal meaning, cycle time was scaled to *percentage of luteal phase elapsed* and *percentage of follicular phase elapsed*. Previous methods count days from menses onset/ovulation and assume a consistent 14-day luteal phase (Schmalenberger et al., 2021), overlooking between- and within-person variations in phase lengths (Bull et al., 2019; Fehring, Schneider, & Raviele, 2006). The luteal phase was defined from day after estimated ovulation (LH + 2) up to and including the day before the next menses onset. The follicular phase was defined from menses onset up to and including the estimated day of ovulation (LH + 1). Our analyses included 421 cycles, with cycle time scaled from −1 to + 1, where zero marked menses onset. This scaling allowed for precise modeling of outcomes centered on menses. In graphs, the −1 to 0 scale of the luteal phase was rescaled to 0 to 100 to correspond to percentage of luteal phase elapsed and is depicted by ‘%L’; the same was done for the follicular phase (i.e. ‘%F’).

#### Stress and adversity inventory

Participants completed the STRAIN during enrollment (Slavich & Shields, 2018), a 415-question, NIMH-recommended measure assessing exposure to 26 acute life events (e.g. death of relative, job loss, negative health event) and 29 chronic difficulties (e.g. persistent health, work, relationship, or financial problems) across lifetime (see https://www.strainsetup.com). An example question is ‘Was there ever a period of time when you were separated from a parent (or main caregiver) for at least one month before you were 18?’ The total possible lifetime stressor exposure count is 166 (range: 0–166).

We calculated counts of total lifetime, pre-menarche, post-menarche, lifetime interpersonal loss (e.g. parental divorce, neglect, loss of a loved one), and lifetime physical danger stressor exposures (i.e. personal injury, such as breaking a leg, instances of physical and sexual abuse) based on reported stressor timings. The STRAIN evaluates everyday stressors and events categorized as traumas by the SCID-5 post-traumatic stress disorder trauma screen (First et al., 2015; Slavich & Shields, 2018). Pre-menarche and post-menarche stressor counts were based on each participant's age of menarche. The STRAIN has excellent test-retest reliability (*r*_icc_ = 0.936 for total count of stressor exposure), concurrent and discriminant validity, and has been shown to predict psychological, biological, and clinical outcomes (Banica, Sandre, Shields, Slavich, & Weinberg, 2022; Cazassa, Oliveira, Spahr, Shields, & Slavich, 2019; Mayer et al., 2023; Murphy et al., 2023; Sturmbauer, Shields, Hetzel, Rohleder, & Slavich, 2019).

#### Negative affective symptoms and suicidal ideation

Participants completed the Daily Record of Severity of Problems (DRSP; Endicott, Nee, & Harrison, 2006), a 21-item, 6-point scale (*not at all* to *extreme*) capturing symptoms of PMDD. Current analyses included items assessing (1) depressed mood, (2) hopelessness, (3) worthlessness/guilt, (4) anxiety, (5) mood swings, (6) rejection sensitivity, (7) anger/irritability, and (8) interpersonal conflict. We calculated a daily average of these *core affective* symptoms of DSM-5 PMDD (American Psychiatric Association, 2013), termed *negative affect*. The study focused on cyclical *emotional* symptoms, so we did not use the mean *total* DRSP score, which would include cognitive, behavioral, and physical symptoms. The reliability of change (R_c_; Revelle, 2023) coefficient for the eight DRSP items was 0.80 indicating reliable within-person covariation. Due to missing data at the survey-level rather than the item-level, means are utilized instead of sums.

Repeated measures correlation coefficients between the eight DRSP items (forming negative affect; Bakdash & Marusich, 2022; Bland & Altman, 1995a, 1995b) varied widely, ranging from 0.27 to 0.70. This prompted additional exploratory analyses to investigate cyclicity of individual DRSP symptoms and identify those driving changes in negative affect. Analyzing effects at the individual symptom-level also helps to inform future research on potential mechanisms specific to each symptom.

Daily surveys also assessed SI severity. Participants rated their past-24-hr agreement to items on a 5-point scale, (*not at all* to *extremely*). Mean daily values of items 1, 9, and 19 of the Adult Suicidal Ideation Questionnaire, as well as ‘I wished I could go to sleep and not wake up’, constituted composite passive SI (Reynolds, 1991) with an *R*_c_ of 0.79. Active SI items included items 2, 17, and 25 of the Adult Suicidal Ideation Questionnaire, as well as ‘I wanted to kill myself’(Reynolds, 1991). Active SI was dichotomized due to the low frequency of active SI endorsement and the lack of convergence of hurdle and zero-inflated models. On days when the participant rated any of the above active SI items above a 1 (*not at all*), that day was assigned a value of 1; otherwise, it was assigned a 0. When considered as a continuous variable, these items did exhibit within-person reliability with an *R*_c_ of 0.76.

### Analytic plan

In R version 4.3.2 we conducted preregistered analyses using packages ‘lme4’ (Bates, Mächler, Bolker, & Walker, 2015), ‘lmerTest’ (Kuznetsova, Brockhoff, & Christensen, 2017), and ‘interactions’ (Long, 2021). We tested the moderating effect of stressors (total, pre-menarche, post-menarche, loss, and danger) on the relationship between the cycle and daily symptoms (e.g. negative affect, passive SI, active SI, and individual affective symptoms). Daily symptom scores (level 1) were nested within participants (level 2). All models included random intercepts and slopes to capture individual differences in symptom trajectories.

To flexibly model nonlinear symptom patterns across the cycle, we used polynomial growth models (Hastie, 2017; Kennedy & Gentle, 2021). These models utilized orthogonal polynomials of cycle time (−1 to +1), reducing multicollinearity between the polynomials, and enabling the inclusion of higher degree random slopes. We assessed the fit of different degree polynomial time (quartic, cubic, quadratic, and linear) in fixed and random effects for predicting outcomes without moderators, employing likelihood ratio tests to determine the best fit. Fixed and random effects of time retained in final models are in results tables (Tables 2, online Supplementary S1-S5).

We tested moderation hypotheses by including stressor exposure (level 2; z-scored) and its interactions with time. The models included age at enrollment (level 2; *z*-scored) to account for the positive relationship between age and stressor count, as well as daily SSRI use (level 1; binary) given its efficacy in treating PMDD (Marjoribanks et al., 2013; Roca et al., 2002; Steinberg, Cardoso, Martinez, Rubinow, & Schmidt, 2012). Sensitivity analysis covaried the interactions between time trends and daily SSRI use. Additionally, we conducted an exploratory analysis to predict daily physical pain from the interactions between time trends and lifetime stressors to consider potential confounding effects of stress on physical symptoms across the cycle.

We used the Johnson–Neyman technique (‘interactions’ package; Long, 2021) on each significant interaction to determine the range of moderator values where stressors significantly influenced cyclicity. Model-implied value graphs display the 75th, 50th, and 25th percentiles of stressor exposures. Graphs of person-mean-centered outcomes across tertiles of stressor exposure are in the supplement.

## Results

### Participants

The final analysis included 6187 daily ratings from 114 participants. Each contributed an average of 54.3 observations (s.d. = 39.9; min = 9; max = 283). The range is due to fewer daily ratings from those still in the trial at the time present analyses were conducted, and more from participants completing the trial during COVID-19-related pauses. Descriptives for demographics, psychiatric diagnoses, and stressor exposure are in Table 1.

**Table 1.** Demographics, stressor exposure, and baseline clinical categories in participants (*N* = 114)

Age in years, *mean* (s.d.)	26.8 (5.3)	
Age at menarche in years, *mean* (s.d.)	11.9 (1.5)	
SSRI use (any use during analyzed study period), *N* (%)	48 (42.1)	
Race and ethnicity, *N* (%)		
African American/Black and Hispanic/Latinx	1 (0.9)	
African American/Black and Non-Hispanic/Latinx	10 (8.8)	
American Indian and Hispanic/Latinx	1 (0.9)	
Asian and Non-Hispanic/Latinx	13 (11.4)	
White and Hispanic/Latinx	10 (8.8)	
White and Non-Hispanic/Latinx	59 (51.8)	
More than one race and Hispanic/Latinx	4 (3.5)	
More than one race and Non-Hispanic/Latinx	7 (6.1)	
Unknown race and Hispanic/Latinx	5 (4.4)	
Unknown race and Non-Hispanic/Latinx	3 (2.6)	
Unknown race and unknown ethnicity	1 (0.9)	
Marital status, *N* (%)		
Single	25 (21.9)	
Married/partnered	88 (77.2)	
Unknown	1 (0.9)	
Education, *N* (%)		
High school degree, GED, or trade school	6 (5.3)	
Some college or 2-year degree	26 (22.8)	
4-year college degree	48 (42.1)	
Post-graduate work	33 (28.9)	
Unknown	1 (0.9)	
Income, *N* (%)		
<$ 15 000	14 (12.3)	
$ 15 000-$ 34 999	30 (26.3)	
$ 35 000-$ 79 999	42 (36.8)	
$ 80 000-$ 100 000	8 (7.0)	
>$ 100 000	17 (14.9)	
Unknown	3 (2.6)	
Sexual orientation, *N* (%)		
Homosexual (lesbian, gay)	11(9.6)	
Bisexual	35 (30.7)	
Heterosexual	54 (47.4)	
Queer/questioning/exploring/other	13 (11.4)	
Unknown	1 (0.9)	
Baseline clinical categories^a^, *N* (%)		
Any current depressive disorder	74 (64.9)	
Any current anxiety disorder	71 (62.3)	
Any current obsessive-compulsive disorder	9 (7.9)	
Any current substance use disorder	17 (14.9)	
Any current eating disorder	11 (9.6)	
Any current trauma-related disorder	32 (28.1)	
Lifetime suicidal behavior (aborted, interrupted, or full attempt)	63 (55.3)	
Stressors^b^	*mean* (s.d.)	*Min,Max*
Lifetime stressors	26.4 (12.7)	4,63
Lifetime physical danger stressors	5.4 (2.9)	0,25
Lifetime interpersonal loss stressors	5.5 (4.8)	0,12
Pre-menarche stressors	5.1 (3.5)	0,16
Post-menarche stressors	12.9 (5.5)	2,25

a *N* does not sum to the sample of *N* = 114 since disorders are often co-morbid.

b For each participant, pre-menarche and post-menarche stressor counts do not sum to their lifetime stressor counts because the STRAIN only assesses age of stressor exposure for the most severe stressor when a stressor has occurred multiple times. The analysis of pre-menarche and post-menarche stressors only includes the counts for stressors for which there is explicit age information.

### Model specification and random effects

To address non-normally distributed residuals and fulfill model assumptions, we applied natural logarithm transformations to negative affect, passive SI, pain, and all DRSP items except depressed mood, with estimates presented on the log scale in tables. To address heteroscedastic residuals of models predicting passive SI, mood swings, and anger/irritability, we utilized a robust sandwich variance-covariance estimator from the ‘clubSandwich’ R package (Pustejovsky, 2023). Employing the original ‘CR0’ sandwich estimator due to our large sample size (Liang & Zeger, 1986), we adjusted for heteroscedasticity to yield standard errors less sensitive to violations of homoscedasticity (Pustejovsky, 2023). For these outcomes, confidence intervals and *p*-values were calculated using robust sandwich estimates for the variance-covariance matrices of the models (Tables 2, online Supplementary S1-S5).

**Table 2.** Lifetime stressors predict affective symptom cyclicity

	log(Negative Affect)			log(passive suicidal ideation)			Active suicidal ideation		
*Predictors*	*Estimates*	*CI* (*95%*)	*p-value*	*Estimates*	*CI* (*95%*)	*p-value*	*Log-Odds*	*CI* (*95%*)	*p-value*
Intercept	0.66	0.60–0.73	**<0.001**	0.42	0.35–0.48	**<0.001**	−1.07	−1.58 – −0.56	**<0.001**
Linear cycle time	−0.74	−1.71–0.23	0.135	−0.49	−1.64–0.66	0.404	−2.70	−11.36–5.96	0.541
Quadratic cycle time	−1.48	−2.23 – −0.72	**<0.001**	−0.94	−1.72 – −0.15	**0.019**	−7.35	−14.61 – −0.09	**0.047**
Cubic cycle time	0.25	−0.30–0.80	0.375	0.04	−0.68–0.75	0.919	0.32	−5.43–6.07	0.914
Quartic cycle time	1.46	0.92–2.01	**<0.001**	1.13	0.48–1.79	**<0.001**	7.26	1.60–12.92	**0.012**
Stressors (sample standardized)	0.05	−0.01–0.11	0.100	0.04	−0.02–0.10	0.217	0.48	−0.01–0.98	0.057
Age (sample standardized)	−0.04	−0.10–0.02	0.209	−0.05	−0.12–0.02	0.163	−0.29	−0.78–0.21	0.255
SSRI	−0.05	−0.10–0.01	0.087	−0.04	−0.10–0.01	0.127	−0.57	−1.08 – −0.06	**0.028**
Linear cycle time × stressors	−0.11	−1.18–0.95	0.836	−0.46	−1.66–0.74	0.453	0.66	−8.14–9.47	0.882
Quadratic cycle time × stressors	−0.76	−1.58–0.06	0.070	−0.79	−1.65–0.07	0.073	−7.97	−15.18 – −0.75	**0.030**
Cubic cycle time × stressors	−0.09	−0.71–0.53	0.775	0.04	−0.73–0.80	0.920	3.05	−2.99–9.10	0.322
Quartic cycle time × stressors	0.06	−0.55–0.67	0.848	0.12	−0.54–0.77	0.726	−3.09	−9.03–2.84	0.307
**Random effects**									
*σ*^2^	0.08			0.08			3.29		
*τ*_00_	0.10 _id_			0.11 _id_			6.05 _id_		
*τ*_11_	13.40 _linear cycle time_			21.32 _linear cycle time_			594.25 _linear cycle time_		
	6.01 _quadratic cycle time_			6.22 _quadratic cycle time_			266.37 _quadratic cycle time_		
*ρ*_01_	0.02			0.02			−0.21		
	0.04			0.09			0.01		
ICC	0.57			0.58			0.65		
N	114 _id_			114 _id_			114 _id_		
Observations	6187			6175			6175		
Marginal *R*^2^/conditional *R*^2^	0.027 / 0.580			0.021 / 0.585			0.038 / 0.661		

All random slopes for time trends were included in initial models and were retained based on likelihood ratio tests. Intraclass correlations for symptom outcomes ranged from 0.57–0.65, indicating approximately 60% of variance was due to between-person differences (Table 2).

### Effects of cumulative life stressors on MCAC

**Figure 1.**
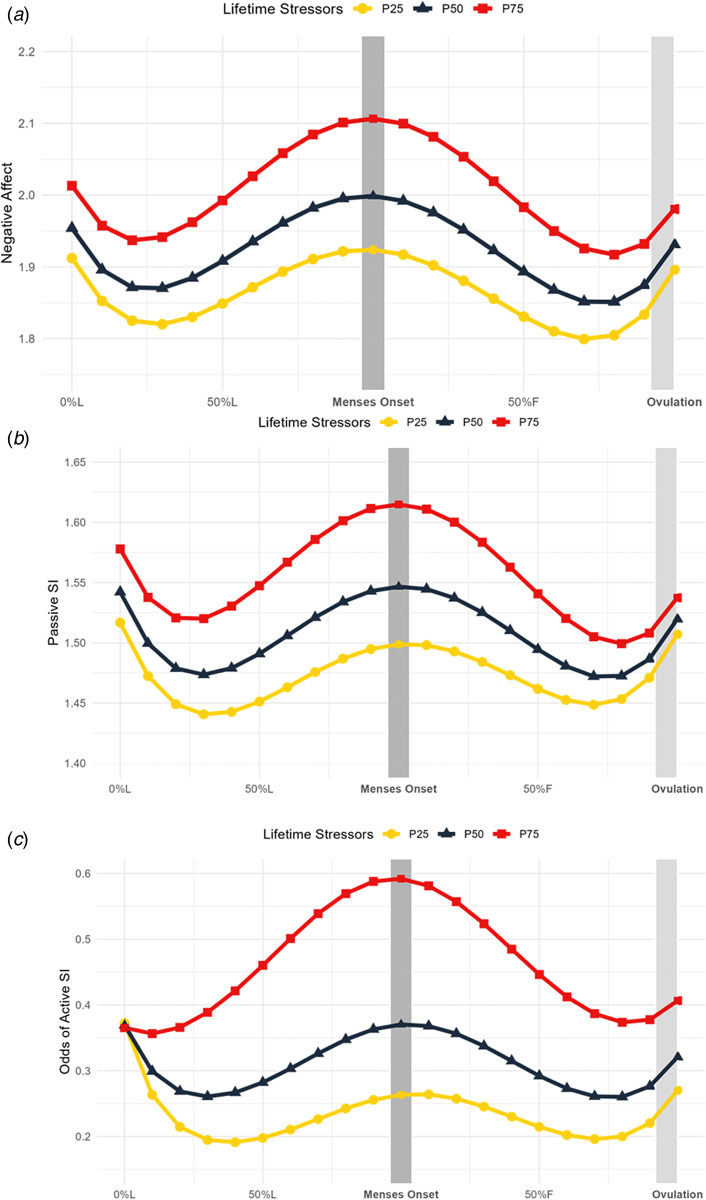
Lifetime stressors predict symptom trajectories across the menstrual cycle. Model-implied values of symptom trajectories across the menstrual cycle by number of lifetime stressors where squares represent more 75th percentile of number of stressors in the sample (34 stressors; P75), triangles represent 50th percentile (23 stressors; P50), and circles represent 25th percentile (15 stressors; P25). L, luteal phase; F, follicular phase. (A) Daily negative affect is a daily mean score of the core emotional symptoms of the daily record of severity of problems (rated from 1 = ‘Not at All’ to 6 = ‘ Extremely’). Marginal significance (*p* = 0.072) in the interaction between lifetime stressors and menstrual cycle time at > 15 stressors (outside Johnson–Neyman interval [− 18 143.50, 15.65]) (B) Daily passive SI is a daily mean score of items 1, 9, and 19 of the Adult Suicidal Ideation Questionnaire, as well as ‘I wished I could go to sleep and not wake up’ (rated from 1 (not at all) to 5 (extremely)). Marginal significance (*p* = 0.076) in the interaction between lifetime stressors and menstrual cycle time at >22 stressors (outside Johnson–Neyman interval [− 12 632.59, 22.52]). (C) Active SI items included items 2, 17, and 25 of the Adult Suicidal Ideation Questionnaire, as well as ‘I wanted to kill myself’. On days when the participant rated any of the above active SI items greater than a 1 (not at all), that day was assigned a value of 0 for active SI; otherwise, they were assigned a value of 1. Odds of active SI were predicted by the number of lifetime stressors. Significance (*p* < 0.05) in the interaction between lifetime stressors and menstrual cycle time at >26 stressors (of outside Johnson–Neyman interval [−82.21, 26.01]).

Consistent with hypotheses that greater lifetime stressors would predict stronger cyclical symptom changes, we observed significant (for active SI) or marginally significant (for negative affect and passive SI) interactions between stressors and quadratic cycle time (Table 2). Greater stress exposure was associated with a stronger increase from the early luteal phase to menses onset, followed by a steeper decline from menses onset to the late follicular phase (Fig. 1, online Supplementary S3).

Johnson–Neyman intervals identified the range of values where stressors significantly influenced the association between cycle time and symptoms. When predicting active SI, the interaction between quadratic time and lifetime stressors reached significance at >26 stressors (significance outside Johnson–Neyman interval [−82.21, 26.01]). Approximately half of the sample (*n* = 55, 48%) experienced more than 26 stressors. When predicting negative affect, the interaction between quadratic time and lifetime stressors reached marginal significance (*p* = 0.072) at > 15 stressors (outside Johnson–Neyman interval [−18 143.50, 15.65]). Seventy-eight percent of the sample experienced more than 15 stressors. For passive SI, the interaction between quadratic time and lifetime stressors reached marginal significance (*p* = 0.076) at >22 stressors (outside Johnson–Neyman interval [−12 632.59, 22.52]). Approximately half of the sample (*n* = 62; 54%) experienced more than 22 stressors. For marginally significant outcomes (e.g. negative affect and passive SI), the Johnson–Neyman interval was calculated at the value of the marginal *p*-values, rather than α = 0.05, reflecting the interaction's marginal significance.

### Effects of pre-menarche and post-menarche stressor exposure on MCAC

We hypothesized that greater pre-menarche stressors, compared to post-menarche stressors, would be associated with greater cyclical increases in negative affect, passive SI, and active SI (online Supplementary Table S1, online Supplementary Figure S1). We observed a significant interaction between pre-menarche stressors and cubic time in predicting active SI, but no other significant moderations by pre-menarche or post-menarche stressors. Increased pre-menarche stressors was associated with an earlier and steeper luteal symptom increase (online Supplementary Figure S1, S4). This interaction reached significance at ≥11 pre-menarche stressors (significance outside Johnson–Neyman interval [0.28, 10.41]); however, just 6.1% of the sample experienced more than 11 pre-menarche stressors (*n* = 7).

### Effects of interpersonal loss and physical danger stressors on MCAC

**Figure 2.**
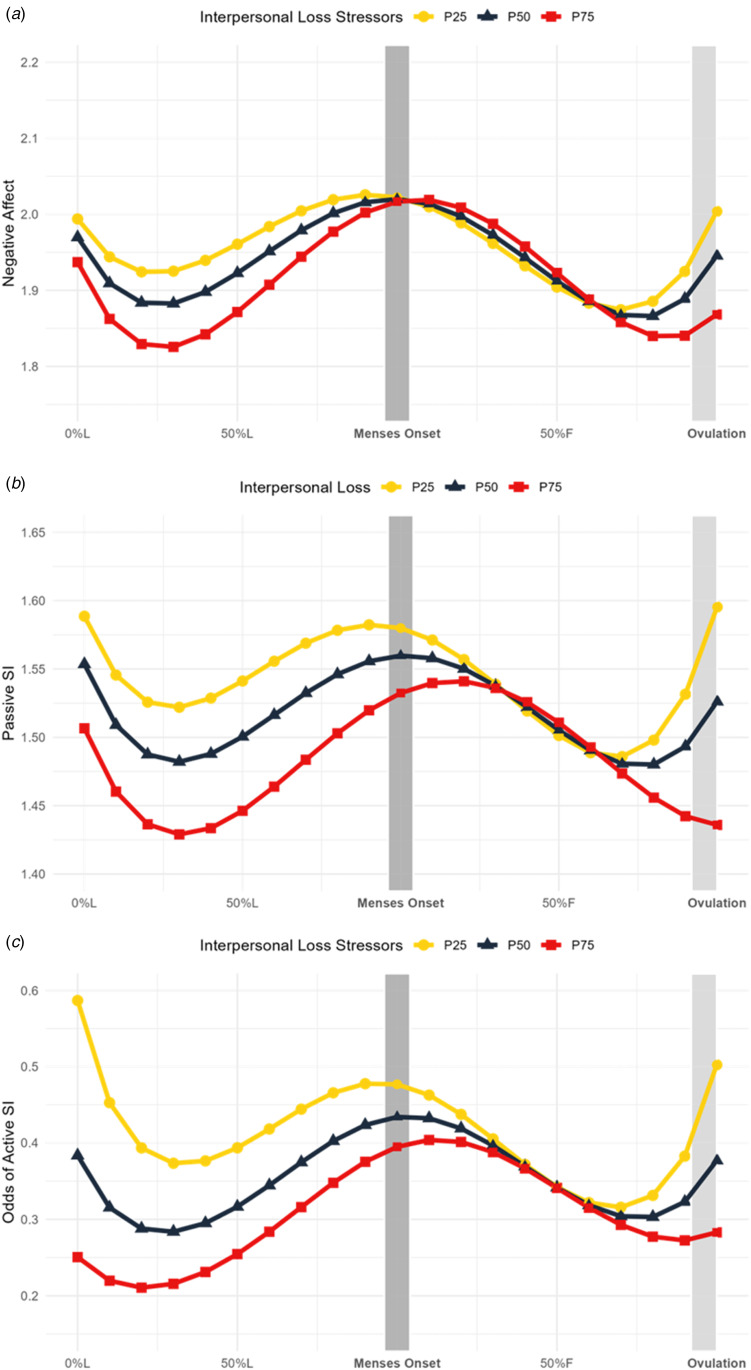
Interpersonal loss stressors predict symptom trajectories across the menstrual cycle. Model-implied values of symptom trajectories across the menstrual cycle by number of interpersonal loss stressors where squares represent more 75th percentile of number of loss stressors in the sample (7.75 stressors; P75), triangles represent 50th percentile (5 stressors; P50), and circles represent 25th percentile (3 stressors; P25). L, luteal phase; F, follicular phase. (A) Daily negative affect is a daily mean score of the core emotional symptoms of the daily record of severity of problems (rated from 1 = ‘Not at All’ to 6 = ‘ Extremely’). Significance (*p* < 0.05) in the interaction between menstrual cycle time and loss stressors at > 3 stressors (outside Johnson–Neyman interval [−34.35, 3.64]). (B) Daily passive SI is a daily mean score of items 1, 9, and 19 of the Adult Suicidal Ideation Questionnaire, as well as ‘I wished I could go to sleep and not wake up’ (*rated from* 1 (not at all) to 5 (extremely)). Significance (*p* < 0.05) in the interaction between menstrual cycle time and loss stressors at >7 stressors (outside Johnson–Neyman interval [0.43, 6.83]). (C) Active SI items included items 2, 17, and 25 of the Adult Suicidal Ideation Questionnaire, as well as ‘I wanted to kill myself’. On days when the participant rated any of the above active SI items greater than a 1 (not at all), that day was assigned a value of 0 for active SI; otherwise, they were assigned a value of 1. Significance (*p* < 0.05) in the interaction between menstrual cycle time and loss stressors at > 5 stressors (outside Johnson–Neyman interval [−2.18, 5.55]). Odds of active SI were predicted by the number of loss stressors.

We hypothesized that danger stressors would predict more pronounced affective cyclicity than loss stressors. Instead, loss was the more useful moderator (online Supplementary Table S2). Loss exposure moderated quadratic and cubic cycle change in negative affect. Specifically, greater loss stressors predicted a steeper symptom increase from the mid-to-late luteal phase, followed by a steeper decline from menses to the late follicular phase. This transition from increasing to decreasing symptoms also occurred later in the cycle for those with higher loss exposure (Fig. 2, online Supplementary S5). Interestingly, greater loss was not associated with greater mean symptoms (Fig. 2, online Supplementary S5). When predicting negative affect, the interaction between cubic time and loss stressors became significant at >3 stressors (outside Johnson–Neyman interval [−34.35, 3.64]); 71.1% of the sample experienced more than 3 loss stressors (*n* = 81).

We observed a similar pattern for passive SI, with an interaction between cubic time and loss stressors (Fig. 2, online Supplementary S5). This interaction reached significance at >7 stressors (outside Johnson–Neyman interval [0.43, 6.83]); 25.4% of the sample experienced more than 7 loss stressors (*n* = 29).

When predicting active SI, we found an interaction between quadratic time and loss stressors (online Supplementary Table S2). The trajectory of active SI symptom cyclicity moderated by loss stressors is depicted in Fig. 2, with person-mean centered plots stratified by loss level in online Supplementary Figure S5. This interaction reached significance at >5 stressors (outside Johnson–Neyman interval [−2.18, 5.55]); 44.7% of the sample experienced more than 5 loss stressors (*n* = 40).

### SSRI sensitivity analysis

We augmented hypothesis 1 models with interactions between SSRI use and time trends (online Supplementary Table S3). These were not statistically significant and did not reduce the significance of stressor by time trend interactions (online Supplementary Table S3, Figure S2).

### Exploratory analysis: predicting physical pain cyclicity from stressor exposure

To examine whether the moderating effects of stressor exposure on cyclical affective symptoms were confounded by similar effects on daily physical pain (e.g. dysmenorrhea), we predicted physical pain from the interaction of cycle time trends and lifetime stressors (online Supplementary Table S4). These interactions did not significantly predict physical pain, suggesting that the influence of stressor exposure on symptom cyclicity is not due to effects on physical pain.

### Exploratory analysis: predicting individual DRSP symptom cyclicity from stressor exposure

**Figure 3.**
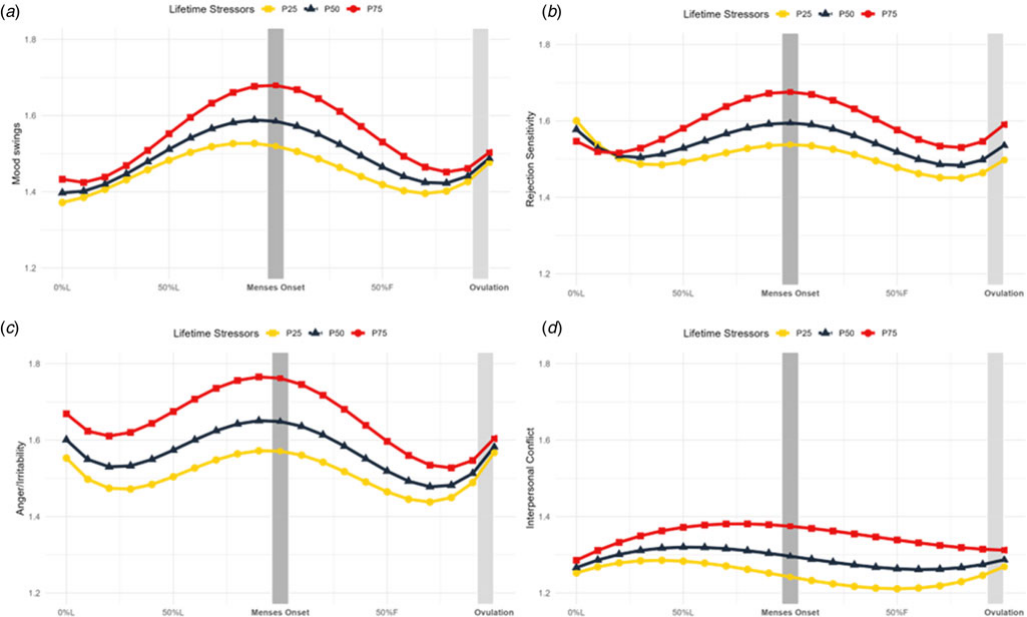
Lifetime stressors predict mood swings, rejection sensitivity, anger and irritability, and interpersonal conflict trajectories across the menstrual cycle. Model-implied values of symptom trajectories across the menstrual cycle by number of lifetime stressors where squares represent more 75th percentile of number of stressors in the sample (34 stressors; P75), triangles represent 50th percentile (23 stressors; P50), and circles represent 25th percentile (15 stressors; P25). L, luteal phase; F, follicular phase. (A) Daily mood swings (rated from 1 = ‘Not at All’ to 6 = ‘ Extremely’) across the menstrual cycle are predicted by number of lifetime stressors. Significance (*p* < 0.05) in the interaction between menstrual cycle time and stressors at > 13 stressors (outside Johnson–Neyman interval [−98.05, 12.61]). (B) Daily rejection sensitivity (rated from 1 = ‘Not at All’ to 6 = ‘ Extremely’) across the menstrual cycle are predicted by number of lifetime stressors. Marginal significance (*p* = 0.079) in the interaction between lifetime stressors and menstrual cycle time at >22 stressors (inside Johnson–Neyman interval [22.40, 731.81]). (C) Daily anger and irritability (rated from 1 = ‘Not at All’ to 6 = ‘ Extremely’) across the menstrual cycle are predicted by number of lifetime stressors. Significance (*p* < 0.05) in the interaction between menstrual cycle time and stressors at > 18 stressors (outside Johnson–Neyman interval [−455.46, 18.05]). (D) Daily interpersonal conflict (rated from 1 = ‘Not at All’ to 6 = ‘ Extremely’) across the menstrual cycle are predicted by number of lifetime stressors. Significance (*p* < 0.05) in the interaction between menstrual cycle time and stressors at > 31 stressors (outside Johnson–Neyman interval [1.84, 31.00]).

Lifetime stressor moderation of symptom cyclicity was significant for mood swings, anger/irritability, and interpersonal conflict, and marginally significant for rejection sensitivity (online Supplementary Table S5). It was not a significant moderator of cyclical effects on depressed mood, hopelessness, worthlessness/guilt, or anxiety. For mood swings, anger/irritability, and rejection sensitivity, greater stressors were associated with an increased slope in symptoms luteally, reaching a peak around menses onset, followed by a steep decline in symptoms into the late follicular phase (Fig. 3, online Supplementary S6, S7). For interpersonal conflict, greater stressors were associated with an increased slope in symptoms across the luteal phase and a slower decline follicularly (Fig. 3, online Supplementary S7).

## Discussion

Although prior research identifies psychosocial stressors as a risk factor for MCAC, it has treated stressor exposure as a unitary construct, overlooking the diversity of stressors across different life stages. To address this, we conducted the largest prospective study to date on the impact of stressors across the life course on symptom reactivity to ovarian hormone fluctuations in a psychiatric sample. Results indicate that greater lifetime stressor exposure predicts more severe perimenstrual affective changes.

### Association between lifetime stressor exposure and affective reactivity to the cycle

Greater lifetime stressor exposure predicted more severe perimenstrual worsening of negative affect, passive SI, and active SI. This effect was significant for active SI, and marginally significant for passive SI and negative affect. While fixed effects reveal that more lifetime stressors predicted more severe symptom trajectories, random effects of cycle time indicate significant variability in MCAC between individuals, consistent with prior longitudinal studies (Eisenlohr-Moul et al., 2020, 2016; Ross et al., 2024). These results align with cumulative stress models suggesting recent and past stressors have an additive effect (Epel et al., 2018; Evans, Li, & Whipple, 2013), leading to worse health outcomes over time (Slavich & Shields, 2018).

### The impact of lifetime stressors on hormone-related affective symptoms across the reproductive lifespan

These results contribute to literature demonstrating that increased stressful life events influence affective symptoms across hormonal fluctuation periods – an effect that has been observed during the menstrual cycle and other reproductive transitions like puberty, pregnancy, and menopause (Allen et al., 2023; Andersen, Klusmann, Eisenlohr-Moul, Baresich, & Girdler, 2023; Gordon, Rubinow, Eisenlohr-Moul, Leserman, & Girdler, 2016). During the peripubertal transition, hormone increases predict greater affective symptoms in high-stress situations (Andersen et al., 2023). Pregnant patients with greater exposure to adverse childhood events experience a more severe depression trajectory throughout the hormonally dynamic antenatal period (Allen et al., 2023). Finally, across the perimenopause transition, estradiol fluctuation predicts greater rejection sensitivity during psychosocial stress (Gordon et al., 2016).

### Pre-menarche stressors, compared to post-menarche stressors influence symptom reactivity to the cycle

Compared to post-menarche stressors, pre-menarche stressors predicted a more severe symptom trajectory of active SI, but not negative affect or passive SI. Greater pre-menarche stressors were associated with an earlier, steeper luteal increase (online Supplementary Figure S1, S4). However, the trend was only significant at high numbers of pre-menarche stressors, experienced by a small subset of the sample (6.1%, *n* = 7). Early life stress may exacerbate cyclical increases in suicidality in individuals with high stress exposure. Further studies comparing early and later life stressors are necessary to validate these findings, as this study is the first to directly compare stressors from different life stages in the context of affective hormone sensitivity.

### Interpersonal loss stressors, compared to physical danger stressors, influence symptom reactivity to the cycle

Compared to danger stressors, loss stressors predicted more severe perimenstrual worsening of negative affect, passive SI, and active SI. Loss and total lifetime stressors followed a similar pattern, with greater stressors linked to steeper symptom increases from the mid-to-late luteal phase, followed by steeper declines from menses to the late follicular phase (Fig. 2, online Supplementary Table S2). While greater loss exposure was not associated with higher mean symptoms, individuals with more stressors experienced more pronounced cyclic symptom patterns, highlighting the possibility that loss stressors more strongly impact symptomatic responses to cycling hormones compared to general risk of symptoms.

### Exploratory analysis: lifetime stressors and individual DRSP item reactivity to the cycle

We examined whether lifetime stressor exposure predicted individual DRSP symptom trajectories to clarify if stressors predicting negative affect was driven by specific symptoms. Lifetime stressor exposure significantly predicted cyclicity of mood swings, anger/irritability, interpersonal conflict, and was a marginally significant moderator of cyclical worsening of rejection sensitivity (online Supplementary Table S5). In contrast, lifetime stressor exposure did not significantly moderate cyclical effects on depressed mood, hopelessness, worthlessness/guilt, or anxiety. These effects are reminiscent of previous research showing that histories of physical abuse predicted a stronger progesterone effect on mood swings, rejection sensitivity, anger/irritability, interpersonal conflict, and depressed mood, with the smallest effect observed on depressed mood (Eisenlohr-Moul et al., 2016). Further work should investigate how specific stressors, particularly physical abuse, predict cyclical changes in mood swings, anger/irritability, and interpersonal conflict.

These findings suggest that cyclical worsening of negative affect is not driven by a single symptom and reflects a broader phenomenon, with changes in mood instability and interpersonal symptoms more strongly linked to stress than depression and anxiety. Notably, conflict displayed a slower follicular return to baseline, possibly related to the stress generation hypothesis (Hammen, 1991): highly stressed individuals who experience increases in negative affect luteally may generate more new stressors (e.g. conflicts), prolonging the return to affective baseline.

### Strengths, limitations, and future directions

The present study employs an innovative, intensive, within-person design to explore relationships between lifetime stressor exposures and affective symptom change across the menstrual cycle. Strengths include pre-registered hypotheses, a large transdiagnostic sample, prospective data, and direct comparisons between pre- and post-menarche stressors, as well as between interpersonal loss and physical danger stressors. Furthermore, we introduced novel methods by standardizing cycle time between participants and flexibly modeling nonlinear trajectories with multilevel polynomial growth models.

However, several limitations should be acknowledged. Although cycle time was determined using LH-confirmed ovulation dates, not every cycle included in the study had an LH-surge confirmed ovulation date due to participants not being required to ovulation test during portions of the parent trial. Instead, we imputed ovulation timing based on a large normative dataset (600 000 cycles) and known menses-to-menses cycle lengths in our data. Ideally, future studies should confirm ovulation in all cycles using biological methods described in Schmalenberger et al. (2021). Additionally, given the rarefied nature of this high-risk sample and descriptive nature of this paper, we prioritized reducing type 2 errors and did not correct for multiple comparisons. We acknowledge that many of the effects described herein would not survive correction for multiple tests, and some may therefore represent Type I errors.

Our results highlight variability in symptom cyclicity, with all models fitting significantly better with random slopes. To understand individual-level symptom patterns, idiographic modeling should be explored. Furthermore, centering cycle time on ovulation rather than (or in addition to) menses onset may reveal whether the periovulatory window is a period of heightened risk for some. Our findings suggest potential periovulatory peaks in negative affect, passive SI, and active SI, demonstrating the importance of investigating this timeframe. Finally, to deepen understanding of how stressors impact cyclical symptoms, future research should examine *perceived severity* of stressors.

## Conclusion

The present study represents the largest prospective test of the link between cumulative lifetime stressor exposure and MCAC in a psychiatric sample. Results suggest that cumulative lifetime stressor exposure predicts greater cycle-related affective changes. Additional research is needed to elucidate the neurobiology of these stress-related effects.

## Supporting information

Nagpal et al. supplementary material 1 Nagpal et al. supplementary material

Nagpal et al. supplementary material 2 Nagpal et al. supplementary material

Nagpal et al. supplementary material 3 Nagpal et al. supplementary material

Nagpal et al. supplementary material 4 Nagpal et al. supplementary material

## Supplementary material

The supplementary material for this article can be found at https://doi.org/10.1017/S0033291724001661.
