# Supplementary material for: Cumulative stressor exposure predicts menstrual cycle affective changes in a transdiagnostic outpatient sample with past-month suicidal ideation
Source: Psychol Med. 2024 Oct 14;54(13):3624–35. doi: 10.1017/S0033291724001661 (PMC11536118; doi:10.1017/S0033291724001661)
Supplement: Nagpal et al. supplementary material 2 — Nagpal et al. supplementary material [file S0033291724001661sup002.docx]

| 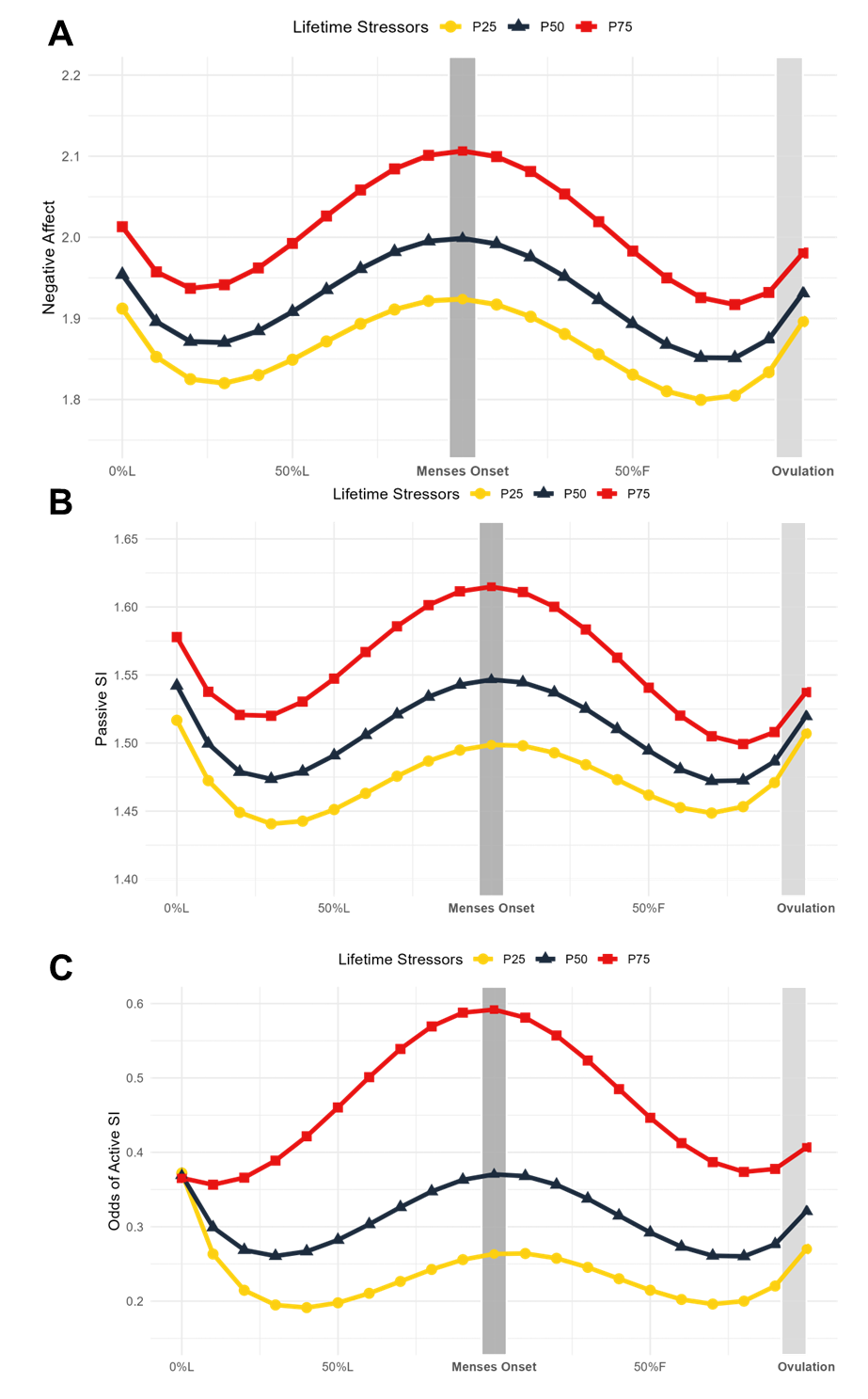 |
| --- |
| **Figure 1. Lifetime stressors predict symptom trajectories across the menstrual cycle.** Model-implied values of symptom trajectories across the menstrual cycle by number of lifetime stressors where squares represent more 75^th^ percentile of number of stressors in the sample (34 stressors) (P75), triangles represent 50^th^ percentile (23 stressors) (P50), and circles represent 25^th^ percentile (15 stressors) (P25). L = luteal phase; F = follicular phase. A) Daily negative affect is a daily mean score of the core emotional symptoms of the Daily Record of Severity of Problems (rated from 1= “Not at All” to 6=” Extremely”). Marginal significance (p = .072) in the interaction between lifetime stressors and menstrual cycle time at > 15 stressors (outside Johnson-Neyman interval [-18143.50, 15.65]) B) Daily passive SI is a daily mean score of items 1, 9, and 19 of the Adult Suicidal Ideation Questionnaire, as well as “I wished I could go to sleep and not wake up” (rated from 1 (not at all) to 5 (extremely)). Marginal significance (p = 0.076) in the interaction between lifetime stressors and menstrual cycle time at $>22$ stressors (outside Johnson-Neyman interval [-12632.59, 22.52]). C) Active SI items included items 2, 17, and 25 of the Adult Suicidal Ideation Questionnaire, as well as “I wanted to kill myself”. On days when the participant rated any of the above active SI items greater than a 1 (not at all), that day was assigned a value of 0 for active SI; otherwise, they were assigned a value of 1. Odds of active SI were predicted by the number of lifetime stressors. Significance ( p < .05) in the interaction between lifetime stressors and menstrual cycle time at > 26 stressors (of outside Johnson-Neyman interval [-82.21, 26.01]) |

| 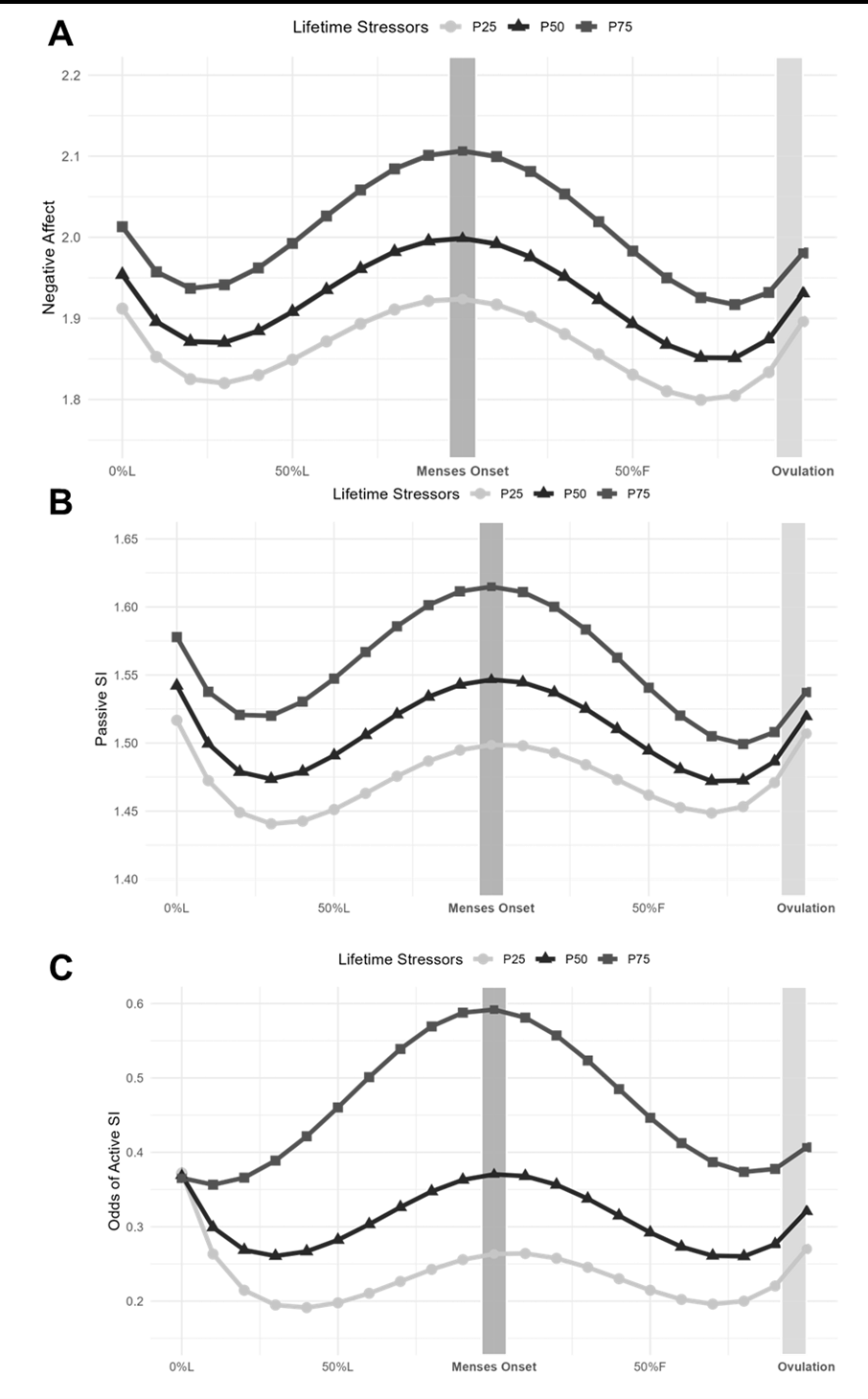 |
| --- |
| **Figure 1. Lifetime stressors predict symptom trajectories across the menstrual cycle.** Model-implied values of symptom trajectories across the menstrual cycle by number of lifetime stressors where squares represent more 75^th^ percentile of number of stressors in the sample (34 stressors) (P75), triangles represent 50^th^ percentile (23 stressors) (P50), and circles represent 25^th^ percentile (15 stressors) (P25). L = luteal phase; F = follicular phase. A) Daily negative affect is a daily mean score of the core emotional symptoms of the Daily Record of Severity of Problems (rated from 1= “Not at All” to 6=” Extremely”). Marginal significance (p = .072) in the interaction between lifetime stressors and menstrual cycle time at > 15 stressors (outside Johnson-Neyman interval [-18143.50, 15.65]) B) Daily passive SI is a daily mean score of items 1, 9, and 19 of the Adult Suicidal Ideation Questionnaire, as well as “I wished I could go to sleep and not wake up” (rated from 1 (not at all) to 5 (extremely)). Marginal significance (p = 0.076) in the interaction between lifetime stressors and menstrual cycle time at $>22$ stressors (outside Johnson-Neyman interval [-12632.59, 22.52]). C) Active SI items included items 2, 17, and 25 of the Adult Suicidal Ideation Questionnaire, as well as “I wanted to kill myself”. On days when the participant rated any of the above active SI items greater than a 1 (not at all), that day was assigned a value of 0 for active SI; otherwise, they were assigned a value of 1. Odds of active SI were predicted by the number of lifetime stressors. Significance ( p < .05) in the interaction between lifetime stressors and menstrual cycle time at > 26 stressors (of outside Johnson-Neyman interval [-82.21, 26.01]) |
