# Supplementary material for: Cumulative stressor exposure predicts menstrual cycle affective changes in a transdiagnostic outpatient sample with past-month suicidal ideation
Source: Psychol Med. 2024 Oct 14;54(13):3624–35. doi: 10.1017/S0033291724001661 (PMC11536118; doi:10.1017/S0033291724001661)
Supplement: Nagpal et al. supplementary material 4 — Nagpal et al. supplementary material [file S0033291724001661sup004.docx]

| **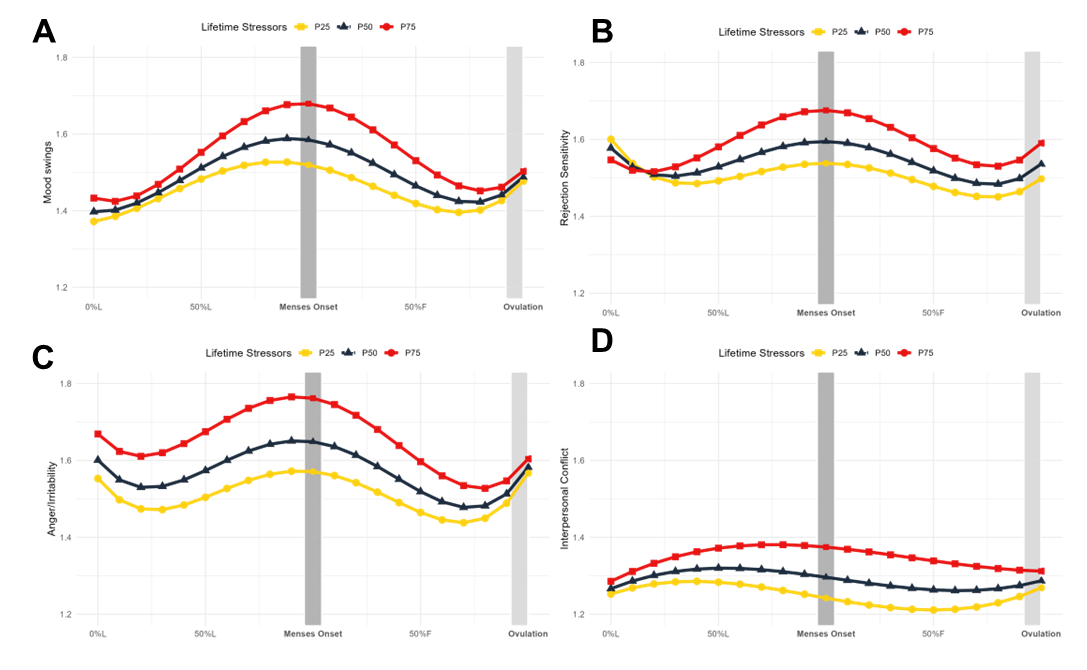** |
| --- |
| **Figure 3. Lifetime stressors predict mood swings, rejection sensitivity, anger and irritability, and interpersonal conflict trajectories across the menstrual cycle.**  Model-implied values of symptom trajectories across the menstrual cycle by number of lifetime stressors where squares represent more 75^th^ percentile of number of stressors in the sample (34 stressors) (P75), triangles represent 50^th^ percentile (23 stressors) (P50), and circles represent 25^th^ percentile (15 stressors) (P25). L = luteal phase; F = follicular phase. A) Daily mood swings (rated from 1= “Not at All” to 6=” Extremely”) across the menstrual cycle are predicted by number of lifetime stressors. Significance (p < .05) in the interaction between menstrual cycle time and stressors at > 13 stressors (outside Johnson-Neyman interval [-98.05, 12.61]). B) Daily rejection sensitivity (rated from 1= “Not at All” to 6=” Extremely”) across the menstrual cycle are predicted by number of lifetime stressors. Marginal significance (p = 0.079) in the interaction between lifetime stressors and menstrual cycle time at $>22$ stressors (inside Johnson-Neyman interval [22.40, 731.81]). C) Daily anger and irritability (rated from 1= “Not at All” to 6=” Extremely”) across the menstrual cycle are predicted by number of lifetime stressors. Significance (p < .05) in the interaction between menstrual cycle time and stressors at > 18 stressors (outside Johnson-Neyman interval [-455.46, 18.05]). D) Daily interpersonal conflict (rated from 1= “Not at All” to 6=” Extremely”) across the menstrual cycle are predicted by number of lifetime stressors. Significance (p < .05) in the interaction between menstrual cycle time and stressors at > 31 stressors (outside Johnson-Neyman interval [1.84, 31.00]). |

| **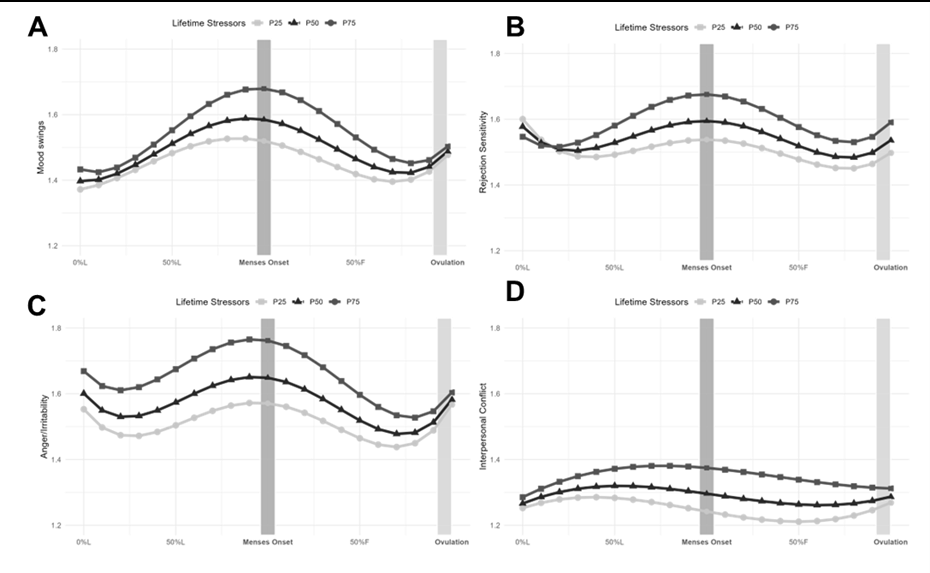** |
| --- |
| **Figure 3. Lifetime stressors predict mood swings, rejection sensitivity, anger and irritability, and interpersonal conflict trajectories across the menstrual cycle.**  Model-implied values of symptom trajectories across the menstrual cycle by number of lifetime stressors where squares represent more 75^th^ percentile of number of stressors in the sample (34 stressors)(P75), triangles represent 50^th^ percentile (23 stressors)(P50), and circles represent 25^th^ percentile (15 stressors)(P25). L = luteal phase; F = follicular phase. A) Daily mood swings (rated from 1= “Not at All” to 6=” Extremely”) across the menstrual cycle are predicted by number of lifetime stressors. Significance (p < .05) in the interaction between menstrual cycle time and stressors at > 13 stressors (outside Johnson-Neyman interval [-98.05, 12.61]). B) Daily rejection sensitivity (rated from 1= “Not at All” to 6=” Extremely”) across the menstrual cycle are predicted by number of lifetime stressors. Marginal significance (p = 0.079) in the interaction between lifetime stressors and menstrual cycle time at $>22$ stressors (inside Johnson-Neyman interval [22.40, 731.81]). C) Daily anger and irritability (rated from 1= “Not at All” to 6=” Extremely”) across the menstrual cycle are predicted by number of lifetime stressors. Significance (p < .05) in the interaction between menstrual cycle time and stressors at > 18 stressors (outside Johnson-Neyman interval [-455.46, 18.05]). D) Daily interpersonal conflict (rated from 1= “Not at All” to 6=” Extremely”) across the menstrual cycle are predicted by number of lifetime stressors. Significance (p < .05) in the interaction between menstrual cycle time and stressors at > 31 stressors (outside Johnson-Neyman interval [1.84, 31.00]). |
